# The Programmable Catalytic Core of 8-17 DNAzymes

**DOI:** 10.3390/molecules29112420

**Published:** 2024-05-21

**Authors:** Fumei Zhang, Weiguo Shi, Lei Guo, Shihui Liu, Junlin He

**Affiliations:** 1 School of Pharmaceutical Sciences, Guizhou University, Guiyang 550025, China; fumeizhang@163.com; 2 Beijing Institute of Pharmacology and Toxicology, Taiping 27, Beijing 100850, China; shiweiguo@bmi.ac.cn (W.S.); guolei@bmi.ac.cn (L.G.)

**Keywords:** DNAzyme, metal ion dependence, catalytic core

## Abstract

8-17 DNAzymes (8-17, 17E, Mg5, and 17EV1) are in vitro-selected catalytic DNA molecules that are capable of cleaving complementary RNAs. The conserved residues in their similar catalytic cores, together with the metal ions, were suggested to contribute to the catalytic reaction. Based on the contribution of the less conserved residues in the bulge loop residues (W^12^, A^15^, A^15.0^) and the internal stem, new catalytic cores of 8-17 DNAzymes were programmed. The internal stem CTC-GAG seems to be more favorable for the DNAzymes than CCG-GGC, while an extra W^12.0^ led to a significant loss of activity of DNAzymes, which is contrary to the positive effect of A^15.0^, by which a new active DNAzyme 17EM was derived. It conducts a faster reaction than 17E. It is most active in the presence of Pb^2+^, with the metal ion preference of Pb^2+^ >> Zn^2+^ > Mn^2+^ > Ca^2+^ ≈ Mg^2+^. In the Pb^2+^ and Zn^2+^-mediated reactions of 17EM and 17E, the same Na^+^- and pH dependence were also observed as what was observed for 17E and other 8-17 DNAzymes. Therefore, 17EM is another member of the 8-17 DNAzymes, and it could be applied as a potential biosensor for RNA and metal ions.

## 1. Introduction

Many kinds of artificially selected ribozymes [1], DNAzymes [2], and aptamers [3] confirm that nucleic acids possess a diversity of natural and artificial functions, except those of intrinsic genetic information carriers and transmitters. Specific tertiary structures and environmental factors (metal ions, small molecules) are supposed to be responsible for new functions. DNAzymes are a kind of in vitro-selected catalytic DNA molecules that are capable of cleaving complementary RNAs, mostly with a metal ion-assisted catalytic mechanism [4,5,6,7]. Among them, DNAzymes 10-23 and 8-17 are the most well known for their efficient catalytic activity and small scale [8]. subsequently, a very similar catalytic motif to that of 8-17 DNAzymes was selected under different selection conditions in several other DNAzymes (Mg5, 17E, and 17EV1) [9,10,11], as shown in Table 1. This repeatedly selected catalytic motif of 8-17 DNAzymes has attracted much attention to obtain an insight into their importance in the catalytic reaction [12], and the roles of individual nucleotides or nucleobases have been explored by various approaches [13,14,15,16,17,18]. A general acid–base catalytic mechanism was suggested, although the details about the roles of individual residues remain to be studied.

In addition to the highly conserved residues (the end loop and C^13^G^14^) related to the catalytic proton transfer [19], the less conserved residues in the large bulge loop (W^12^, A^15^, and A^15.0^) were suggested to be related to the different catalytic metal ion dependences [14]. In our previous research on the adenine residues (A^12^, A^15^, A^15.0^) of 8-17DZ and 17E with functional group modifications, A^12^ and A^15.0^ could also be recognized as the conserved residues at the level of functional groups, and A^15^ could be modified to induce different metal ion dependences and more efficient catalytic reactions [18]. And more DNAzymes with similar catalytic cores have, indeed, been selected recently [20,21,22]. These facts demonstrate that the flexibility of the catalytic loop remains to be explored for more efficient DNAzyme variants.

**Figure 1:**
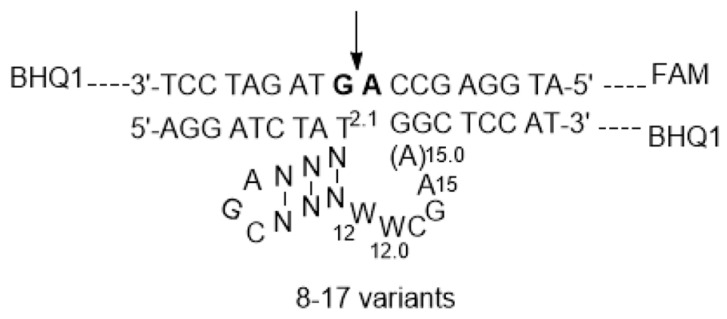
The secondary structure of 8-17 DNAzyme variants, designed to be against a DNA-RNA-DNA substrate with RNA residues in bold letters, W = A or T. The arrow indicates the cleavage site in the substrate. The FAM and BHQ1 groups were attached to the system for signaling of the catalytic reaction.

DNAzyme variants are shown in Figure 1. In the search for new DNAzymes, de novo selection and chemical modifications were the most often used methods. For the 8-17 DNAzymes, a simple replacement of each residue in the catalytic core with the other three canonical residues generally could not lead to a better DNAzyme, but the conserved residues were identified. Meanwhile, our chemical modifications at the level of functional groups in the catalytic core succeeded, and more efficient DNAzymes were obtained [18,23]. From these studies, we thought that the catalytic core would be programmable for better results if it was recognized at the level of the motif. At least two kinds of stems and three large bulge loops could be combined for an effective DNAzyme, except for the highly conserved end loop and G●T wobble pair. Here, a combination of the internal stem and the large loop were programmed for new DNAzymes, and an active DNAzyme was constructed and evaluated for its primary mechanistic properties.

## 2. Results and Discussion

### 2.1. The Positive Effect of the Internal Stem in 8-17 DNAzymes

In the present evaluation system, DNAzymes were designed to be against a DNA-RNA-DNA chimeric substrate (8-17S), forming an active catalytic complex in the presence of divalent metal ions (Figure 1). The fluorescence intensity was used as the indicator of the catalytic reaction, as these DNAzymes have been coupled with fluorescence imaging for the intracellular detection of biologically important metal ions and RNAs [24,25,26,27]. In the complex, the substrate was labelled with a fluorescent molecule (FAM) and a black hole quencher (BHQ1) at the 5′- and 3′-end, respectively, and the DNAzymes were labelled with BHQ1 at the 3′-end. FAM at the 5′-end of the substrate is located near the quencher molecule (BHQ1) at the 3′-end of the DNAzyme, and no fluorescence signal can be emitted. When the substrate is catalytically cleaved by the DNAzyme, the cleaved product with 5′-FAM is released, and a fluorescence signal can be produced by excitation, indicating the reaction process. The BHQ1 at the 3′-end of the substrate was introduced for the minimization of the background fluorescence of the system. 

Firstly, the four most well-known DNAzymes, 8-17, 17E, Mg5, and 17EV1, were evaluated in the present system. Under single-turnover conditions, Pb^2+^, Zn^2+^, Mn^2+^, Ca^2+^, or Mg^2+^ was used to initiate the reaction, respectively, in the buffer system of 50 mM HEPES (pH 7.27) containing 100 mM Na^+^, as these metal ions were most often used for the selection and evaluation of the four most well-known DNAzymes. Secondly, these metal ions are critically related to biological functions, and Pb^2+^ is especially toxic to children. In addition, Na^+^ was used as a cofactor for the evaluation system, because it was also used in the selection conditions (Table 1), ranging from 25 mM to 1 M. Our experiments demonstrated that Na^+^ had a positive effect on the catalytic reactions of DNAzymes, and 100 mM Na^+^ is most effective for the reaction.

**Figure 2:**
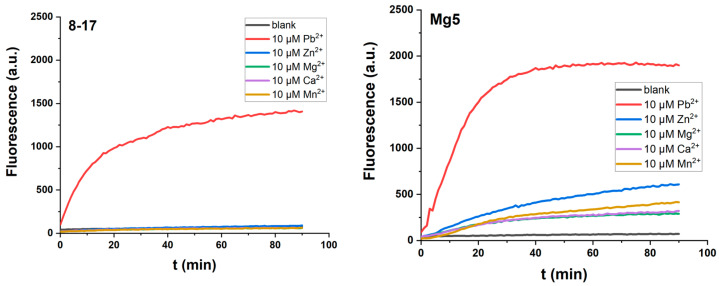

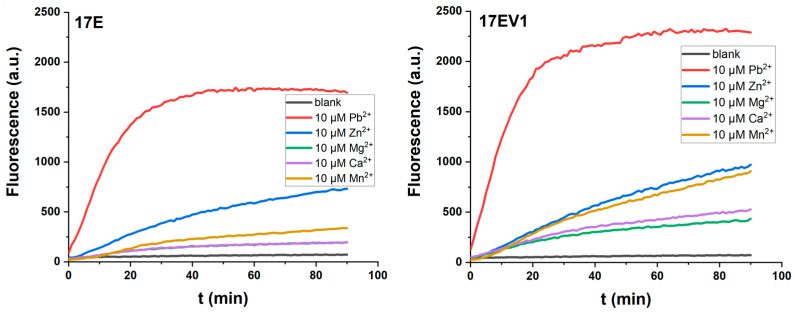
The catalytic reaction of 8-17 DNAzyme variants 8-17, Mg5, 17E, and 17EV1 by fluorescent signaling under single-turnover conditions (0.5 μM DNAzyme and 0.05 μM substrate) in the buffer (50 mM HEPES, pH 7.27) containing 100 mM Na^+^, in the presence of 10 μM Pb^2+^, Zn^2+^, Mn^2+^, Ca^2+^, Mg^2+^, respectively.

As shown in Figure 2, all these DNAzymes exerted a very similar metal ion dependency, which is also reported in the literature (Table 1). They are most active in the presence of Pb^2+^ [28], as shown by them having the fastest increase and the strongest fluorescence signaling during the reaction. Therefore, these DNAzymes have been studied as biosensors for Zn^2+^and Pb^2+^, as well as mRNA and miRNA in living cells and animals [29,30,31], as when they were applied in a cell-mimicking buffer system (50 mM Tris-HCl, pH 7.4, 100 mM NaCl, 50 mM KCl, 1 mM MgCl_2_) [24], only a weak fluorescent signaling was produced (Figure S1).

**Figure 3:**
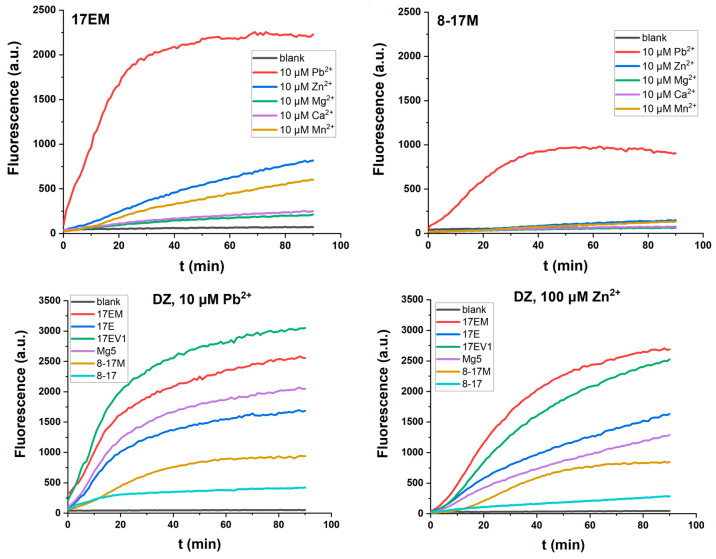
The catalytic reaction of 17EM and 8-17M and a comparison with other 8-17 DNAzymes, indicated by fluorescent signaling under single-turnover conditions (0.5 μM DNAzyme and 0.05 μM substrate) in the buffer (50 mM HEPES, pH 7.27) containing 100 mM Na^+^, in the presence of Pb^2+^, Zn^2+^, Mn^2+^, Ca^2+^, Mg^2+^, and (10 μM), respectively.

Among these DNAzymes, 17EV1 and Mg5 have the same large bulge loop (ACGAA), but 17EV1 always conducts a faster reaction. It is possible that the internal stem CTC-GAG could exert a positive effect in the context of 17EV1. Our previous research on G^11^ in the stem, CCG-CGG^11^, confirmed that it could be modified for a positive effect [23]. Based on these facts, the stem of 17E was replaced by CTC-GAG to obtain a new DNAzyme, 17EM (Table 1), and the same substitution was applied for 8-17 to obtain 8-17M. As shown in Figure 3, the CTC-GAG stem was indeed more favorable for the catalytic reaction of DNAzymes 17EM and 8-17M, when both Pb^2+^- and Zn^2+^-mediated reactions were compared. Furthermore, both DNAzymes had a similar metal ion dependence as other 8-17 DNAzymes, and they were most active in the Pb^2+^-mediated catalytic reaction, when the same concentrations of all five metal ions were compared.

### 2.2. The Effect of the Extra W^12.0^ on DNAzymes

In the case of the bulge loop, it is interesting to note that the extra A^15.0^ exerts a significant positive contribution to the catalytic activity of 17E, Mg5, 17EV1, and 17EM when compared to the 8-17 DNAzyme. Next, 17EV1 and 8-17 were selected for the incorporation of an extra A^12.0^ in the large loop to afford two new DNAzymes, 8-17M01 and 8-17M02, respectively. However, these two enzymes were much less active when evaluated under the same conditions (Figure S2) in the presence of five divalent metal ions (Pb^2+^, Zn^2+^, Mn^2+^, Ca^2+^, or Mg^2+^, respectively). The effect of W^12.0^ (A^12.0^ or T^12.0^) was further tested for these DNAzymes, as shown in Table 2, using 17MM01 to 17MM03 with a 6 nt bulge loop, and 17MM04 to 17MM06 with a 5 nt bulge loop. All the DNAzymes were much less active when tested in the presence of 10 μM divalent metal ions (Figure S2), while some of them still worked in the presence of higher concentrations of metal ions (Figure S3). Similarly, the Pb^2+^ mediated the fastest reaction, and 8-17M01 and 8-17MM5 were more active than the others, probably because they were derived from the large loop (ACGAA) of the most effective DNAzyme, 17EV1. For all the DNAzymes with a 5 nt bulge loop, the effects of A^15.0^ and A^12.0^ were completely different, indicating the limited flexibility of the specific catalytic conformation, in which the interaction network of critical residues and metal ions are defined by surrounding residues. 

From these two kinds of modifications in the catalytic core, an active DNAzyme 17EM was obtained; its catalytic performance was close to that of in vitro-selected DNAzymes (Figure 3), and its catalytic reaction was further evaluated for its primary mechanistic properties.

### 2.3. Thermal Stability and CD Spectra of DNAzyme–Substrate Complex System

**Figure 4:**
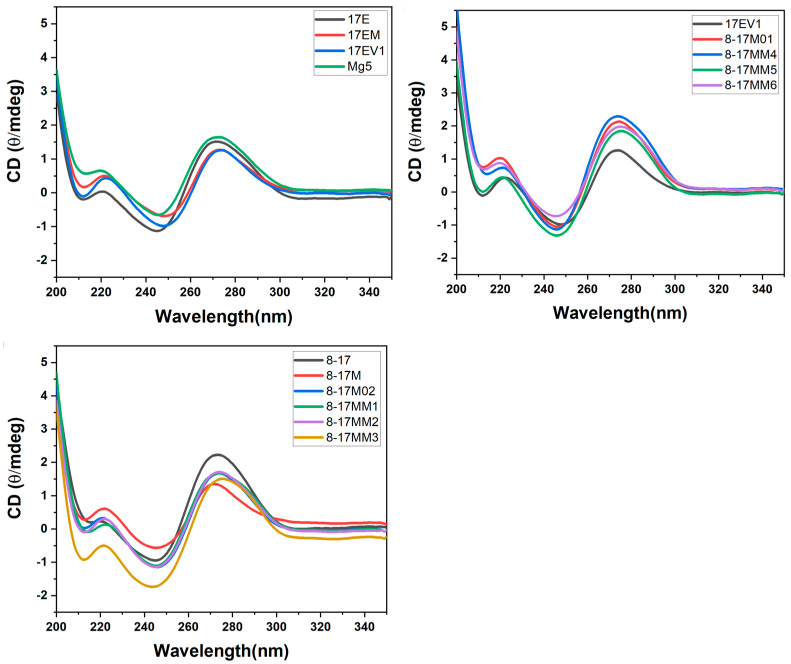
CD spectra of DNAzyme–substrate complex in the buffer system (50 mM HEPES, pH 7.27) containing 100 mM Na^+^ and 2 mM Mg^2+^.

As the DNAzyme–substrate complex formation is a prerequisite for the catalytic reaction, all the DNAzymes were checked for their complex stability in the present system. The full-DNA substrate (D18) was used instead of the DNA-RNA-DNA chimeric substrate to avoid the cleavage reaction and digestion on the substrate during the measurement. The CD spectra of these complexes demonstrated that all the complexes showed the characteristic B-duplex conformation (Figure 4), with the positive lobe around 275 nm, the negative lobe around 250 nm, and a crossover at 260 nm [32]; however, small differences between the complexes could be observed, although the effect of the large loop could not be distinguished from the whole conformation. On the other hand, as shown in Table 2, the similar T_m_ indicated that all the DNAzymes could form a stable complex under the present conditions. The duplex between the recognition arms and the substrate was suggested to be the main stabilizing factor for the complex formation. These data may indicate the local conformational changes caused by the different bulge loop residues, as indicated by their different effects on the catalytic reaction.

### 2.4. The Metal Ion Dependence of 17EM

**Figure 5:**
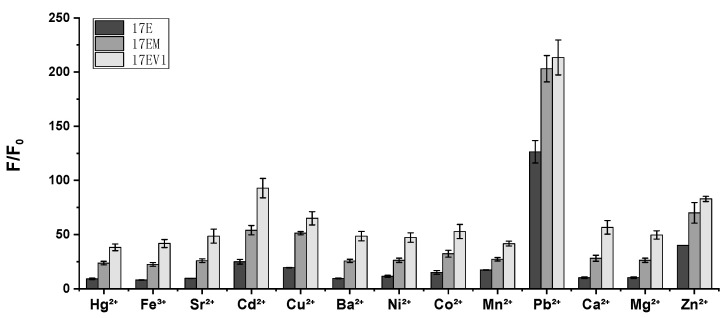
A comparison of DNAzymes 17EM, 17E, and 17EV1 in terms of the effect of multivalent metal ions. The fluorescence intensity increase was recorded (at 40 min) under single-turnover conditions for the reactions of 17EM, 17E, and 17EV1 (0.5 μM) against 8-17S (0.05 μM) in 50 mM HPEPS (pH 7.27) containing 100 mM Na+, and 10 μM of Pb^2+^, Zn^2+^, Ca^2+^, Mn^2+^, Mg^2+^, Hg^2+^, Fe^3+^, Sr^2+^, Cd^2+^, Cu^2+^, Ba^2+^, Ni^2+^, or Co^2+^ was added, respectively, to initiate the reaction.

As described above, 17EM had a similar metal ion dependence to other 8-17 DNAzymes, with a tendency of Pb^2+^ >> Zn^2+^ > Mn^2+^ > Ca^2+^ ≈ Mg^2+^; here, its metal ion dependence was further evaluated in the present system with eight other multivalent metal ions. Under single-turnover conditions, 17EM still had the same metal ion dependence as the other 8-17 DNAzymes (17E and 17EV1), as shown in Figure 5, and they were all most active in the Pb^2+^-mediated reaction. It is well recognized that the contribution of Pb^2+^ is unique in the catalytic reaction of 8-17 DNAzymes in terms of the physicochemical properties of the Pb^2+^- and Pb^2+^-induced global folding of the complex and the cooperative role of Na^+^ [19,33]. The hydrated metal ion Pb^2+^ was suggested to act as the general acid in the catalytic reaction.

### 2.5. pH Dependence of Pb^2+^-Mediated Reaction of 17EM

**Figure 6:**
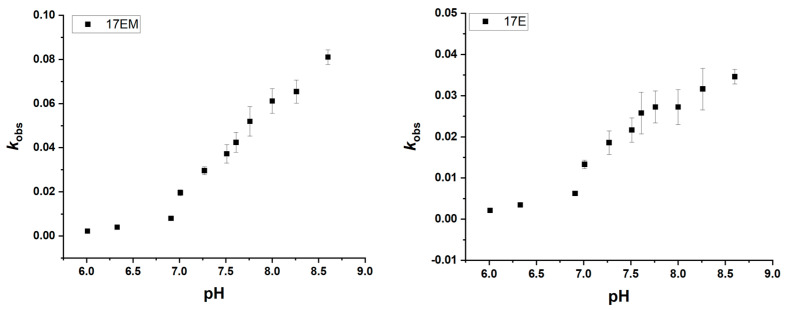
pH dependence of 17EM and 17E under single-turnover conditions. An increase in the fluorescence intensity was recorded for the reactions between 0.5 μM DNAzyme and 0.05 μM substrate in the buffer with different pH values (with 100 mM Na^+^).

For the Pb^2+^-mediated catalytic reaction of 17EM, the pH dependence was investigated. As shown in Figure 6, with an increasing pH, an increase in the rate of fluorescence was observed. The similar linear pH dependence of *k*_obs_ between 17EM and 17E, in the pH range of 6.0–8.6, indicated that these DNAzymes conduct the catalytic reaction by the same general acid–base mechanism [11,34].

### 2.6. The Influence of Sodium Ions on the Catalytic Reactions of 17EM and 17E

**Figure 7:**
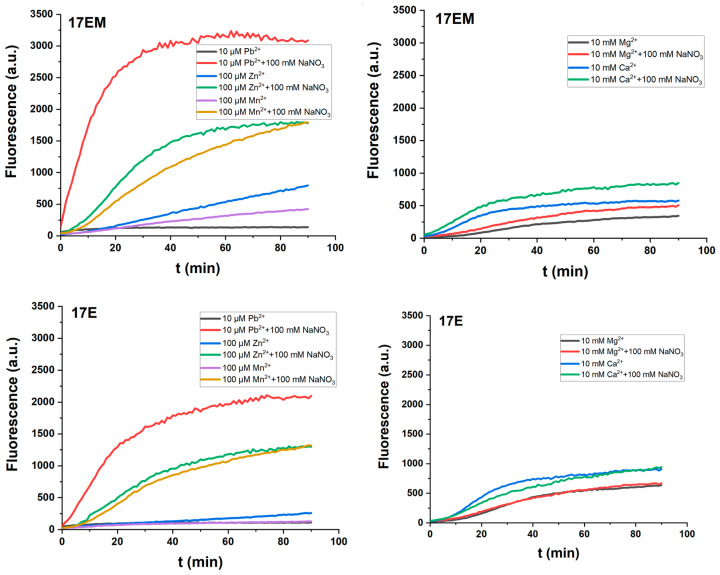
The effect of Na^+^ on the reaction of DNAzymes under single-turnover conditions. An increase in the fluorescence intensity was recorded for the reactions between 0.5 μM DNAzyme and 0.05 μM substrate in the buffer (50 mM HEPES, pH 7.27) containing 100 mM Na^+^, in the presence of 10 μM Pb^2+^, 100 μM Zn^2+^, 100 μM Mn^2+^, 10 mM Ca^2+^, or 10 mM Mg^2+^, respectively.

Next, the effect of Na^+^ on 17EM and 17E was investigated. As shown in Figure 7, the positively cooperative role of Na^+^ is significant in the case of Pb^2+^-, Zn^2+^-, and Mn^2+^-assisted reactions, but not in the case of Ca^2+^ and Mg^2+^. A similar contribution of Na^+^ was also observed for the 8-17 DNAzyme [35]. Na^+^ was supposed to play a promotive role, probably by strengthening the electrostatic interaction within the catalytic residue–metal ion complex [19]. On the other hand, it might indicate that soft and hard metal ions are involved in these DNAzyme-mediated reactions in different ways, due to their different physicochemical properties [36]. 

### 2.7. The Unique Pb^2+^-Mediated DNAzyme Reaction

Multiple roles have been suggested for metal ions in the catalytic reaction of DNAzymes, and especially, roles for 8-17 DNAzymes were suggested, including the structural organization of an active conformation, tuning of nucleobases pKa to an activated form (general base/acid), and direct involvement in the reaction as a general acid/base. From the mutation analysis of the catalytic core, one of the roles of the conserved residues was supposed to form an interaction network with the catalytic metal ion, supporting the specific binding location of metal ions to conduct the cleavage reaction. 

**Figure 8:**
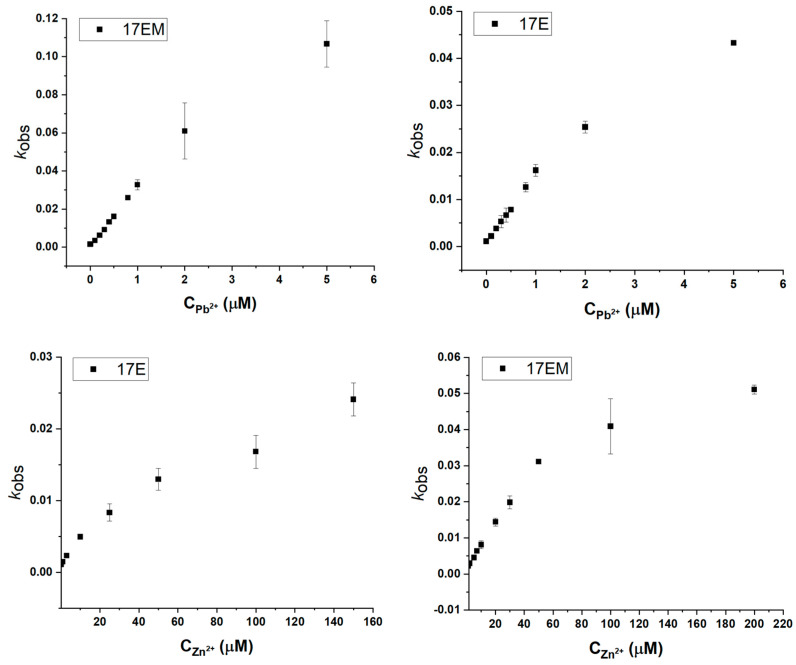
Metal ion concentration dependence of 17EM and 17E under single-turnover conditions. A fluorescence intensity increase was recorded for the reactions between 0.5 μM DNAzyme and 0.05 μM substrate in the buffer (50 mM HEPES, pH 7.27) containing 100 mM Na^+^, Pb^2+^, or Zn^2+^ at different concentrations, added to start the reaction.

The Pb^2+^-dependent catalytic mechanism of DNAzymes has been the focus of many studies. From the dependence of *k*_obs_ on pH, a singe deprotonation in the rate-limiting step of the reaction was suggested for DNAzymes (8-17, 17E). In addition, from MALDI-TOF MS analysis of the cleavage products, it was demonstrated that Pb^2+^-DNAzymes share a two-step mechanism with ribonucleases and RNAzymes [27], while other metal ions run a single-step mechanism, because only Pb^2+^ could catalyze the hydrolysis of the cyclic intermediate, as supposed for ribonucleases and leadzyme. The very similar dependence of *k*_obs_ on the pH and Pb^2+^concentration (Figure 8), as well as the metal ion preference, indicated that 17EM conducts the reaction with the same mechanism as 17E and other 8-17 DNAzymes. 

The information about the catalytic structure and the interaction with metal ions of 8-17 DNAzymes was studied with various approaches, including charge flow experiments [37], contact photo-cross-linking investigations [38], FRET [39], and other methods. These data implied that Zn^2+^ and Mg^2+^ induced a global folding of the complex, while Pb^2+^ does not need a global folding for the cleavage reaction [12]. In other words, Pb^2+^ mediates the catalytic reaction in a unique mode, which is different from other metal ions.

### 2.8. The Detection Limit of Pb^2+^ and Zn^2+^ of 17EM and 17E

**Figure 9:**
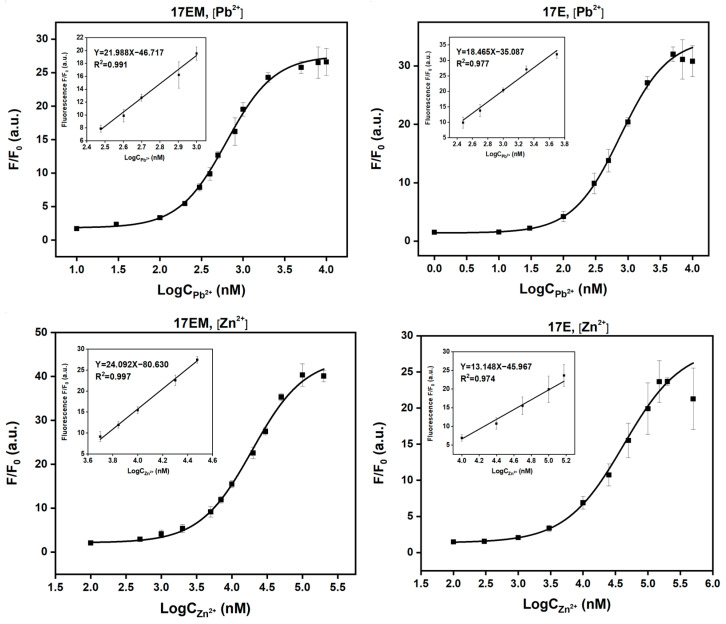
The LOD calculation of Pb^2+^ and Zn^2+^ of 17EM and 17E under single-turnover conditions. A fluorescence intensity increase was recorded for the reactions between 0.5 μM DNAzyme and 0.05 μM substrate in the buffer (50 mM HEPES, pH 7.27) containing 100 mM Na^+^, Pb^2+^, or Zn^2+^ at different concentrations, added to start the reaction.

8-17 DNAzymes have been studied as the biosensors for Pb^2+^, Zn^2+^, and RNAs, both in vitro and in vivo, by combining fluorescent signaling [23,29,30]. This meant that these DNAzymes could accommodate the bulky dye molecules and were compatible with the aquatic biological media and living cells. Based on the similar catalytic performance of 17EM and 17E, we reasoned that 17EM could be recognized as a potential biosensor, too. Here, the limit of detection of 17EM for Pb^2+^ and Zn^2+^ was assayed by the fluorescence signaling method (Figure 9). Under single-turnover conditions, in the HEPES buffer (pH 7.27) containing 100 mM Na^+^, the limit of detection (LOD) of 17EM1 was 182.43 nM for Pb^2+^ and 2.96 μM for Zn^2+^, and the LOD of 17E was 115.52 nM for Pb^2+^ and 5.30 μM for Zn^2+^.

## 3. Materials and Methods

### 3.1. Materials

DNAzyme oligonucleotides labelled with 3′-BHQ1 were purchased from Sangon (Shanghai, China), and the DNA-RNA-DNA chimeric substrate labelled with 5′-FAM and 3′-BHQ1 were purchased from Takala (Dalian, China). The concentrations of all oligonucleotides were determined by UV absorbance at 260 nm and the extinction coefficient by the nearest neighbor method. 

### 3.2. Thermal Stability Measurement

The complexes between DNAzymes and the full-DNA substrate D18 were formed in the HEPES buffer (pH 7.27) containing 100 mM NaNO_3_ and 2 mM Mg^2+^. The thermal stability was measured on an S-1700 (Shimazu, Japan). The above solution was heated at 90 °C for 10 min, and then, it was cooled at a rate of 1 °C/min, and the UV absorbance was recorded simultaneously. The T_m_ values were estimated as the maxima of the first derivatives of the annealing curves, and the error was ±1 °C.

### 3.3. CD Spectra

The DNAzyme–substrate complex solution from the T_m_ measurement was used for CD spectra measurement on a Chieascan Plus (Applied Photophysics, Leatherhead, UK). With a scanning rate of 100 nm/min and a bandwidth of 1 nm, three scans with background extraction were averaged and smoothed.

### 3.4. The Catalytic Reaction

The reaction of the DNAzyme (0.5 μM) against 8-17S (0.05 μM) was assessed under single-turnover conditions in a buffer of 50 mM HPEPS (pH 7.27) with or without 100 mM NaNO_3_; the metal ions were added to initiate the reaction, and fluorescence signal was collected simultaneously on an infinite M1000Pro. For FAM, the *E*x = 490 nm, and *E*m = 525 nm. The single exponential decay function P% = P∞%–C exp [1–*k*_obs_t] was used to calculate the observed rate constants, where P is the fluorescence intensity at time t with background extraction, C is the difference of P% between t = ∞ and t = 0, and P∞ is the endpoint fluorescence intensity at 48 h with 10 μM Pb^2+^ when no change was observed [40]. The data were the averaged result of three independent experiments, with a variation of <20%. 

## 4. Conclusions

8-17 DNAzymes are the most studied DNA molecules for their potential applications and catalytic mechanism. In the search for new DNAzymes, in vitro selection and chemical modifications have been the most often used methods. In our research on more efficient DNAzymes, these catalytic cores are recognized at the level of the motif, and our particular interest is the variable large loops and the internal stems in their catalytic cores. The replacement of stem CCG-CGG with CTC-GAG in 17E and 8-17 DNAzyme led to a positive effect, and a more active DNAzyme 17EM was created. In the large loop, an extra residue W^12.0^ was not favorable for the catalytic reaction of all DNAzymes, which is in contrast to the significant positive contribution of A^15.0^.

17EM conducts a more effective catalytic reaction than 17E does. Both of them have a similar metal ion preference, as well as the same dependence on the pH, metal ion concentration, and sodium ion, indicating that they conduct the reaction through the same catalytic mechanism. Similarly, 17EM could be developed as a biosensor for Pb^2+^, Zn^2+^, and RNA by a combination with various signaling methods, as other 8-17 DNAzymes. 

In the catalytic cores of these 8-17 DNAzymes, the contribution of individual residues and the active conformation are worth further exploration to improve our understanding of the catalytic DNAs and other functional nucleic acids. The Pb^2+^-mediated most active reaction of all the DNAzymes is of particular interest. Based on the present approaches and progress, Pb^2+^ could be involved in the reaction, and due to its unique physicochemical properties, a general acid catalysis and unique folding for Pb^2+^ were proposed.

## Figures and Tables

**Table 1:** In vitro selection conditions for 8-17 DNAzymes and their critical catalytic motifs.

DNAzyme	Selection Conditions	End Loop	Internal Stem	Bulge Loop	Metal Ion Dependence	Ref.
8-17	10 mM MgCl_2_/1 M NaCl, 50 mM Tris-HCl, pH 7.5, 37 °C	AGC	CCG GGC	ACGA	Pb^2+^ >> Mg^2+^, Ca^2+^	[8]
Mg5	0.5 mM Mg^2+^/50 mM histidine, 50 mM Na_3_PO_4_, pH 7.0, 125 mM NaCl, 125 mM KCl, 37 °C	AGC	CCG GGC	ACGAA	Pb^2+^ >> Zn^2+^, Ca^2+^	[9]
17E	100 μM Zn^2+^, 500 mM NaCl, 50 mM HEPES, pH 7.0, 25 °C	AGC	CCG GGC	TCGAA	Pb^2+^ >> Zn^2+^ >> Mn^2+^ > Mg^2+^~Ca^2+^	[10]
17EV1	50 mM MES, pH 6.0, 25 mM NaCl, human serum	AGC	CTC GAG	ACGAA	Pb^2+^ >> Zn^2+^, Mn^2+^ > Ca^2+^, Mg^2+^	[11]
17EM	-	AGC	CTC GAG	TCGAA	Pb^2+^ >> Zn^2+^, Mn^2+^ > Ca^2+^, Mg^2+^	
8-17M	-	AGC	CTC GAG	ACGA	Pb^2+^ >> Mg^2+^, Ca^2+^	

**Table 2:** 8-17 DNAzyme variants with specific recognition arms in this study.

DNAzyme	Sequence (5′-3′)	T_m_^1^
17EV1	agg atc tat CTC AGC GAG ACGAA ggc tcc at-BHQ1	39.8
Mg5	agg atc tat CCG AGC CGG ACGAA ggc tcc at-BHQ1	40.0
17E	agg atc tat CCG AGC CGG TCGAA ggc tcc at-BHQ1	41.6
17EM	agg atc tat CTC AGC GAG TCGAA ggc tcc at-BHQ1	42.2
8-17	agg atc tat CCG AGC CGG ACGA ggc tcc at-BHQ1	39.8
8-17M	agg atc tat CTC AGC GAG ACGA ggc tcc at-BHQ1	40.0
8-17M01	agg atc tat CTC AGC GAG AACGAA ggc tcc at-BHQ1	41.3
8-17MM4	agg atc tat CTC AGC GAG ATCGAA ggc tcc at-BHQ1	41.9
8-17MM5	agg atc tat CTC AGC GAG TACGAA ggc tcc at-BHQ1	41.8
8-17MM6	agg atc tat CTC AGC GAG TTCGAA ggc tcc at-BHQ1	40.2
8-17M02	agg atc tat CTC AGC GAG AACGA ggc tcc at-BHQ1	40.5
8-17MM1	agg atc tat CTC AGC GAG ATCGA ggc tcc at-BHQ1	42.9
8-17MM2	agg atc tat CTC AGC GAG TACGA ggc tcc at-BHQ1	40.0
8-17MM3	agg atc tat CTC AGC GAG TTCGA ggc tcc at-BHQ1	43.9
8-17S	FAM-d(ATGGAGCC)-r(AG)-d(TAGATCCT)-BHQ1	
D18	ATGGAGCCAGTAGATCCT	

^1^ T_m_ was measured for DNAzyme–substrate complexes in HEPES (50 mM, pH 7.27) containing 100 mM Na^+^ and 2 mM Mg^2+^, with a standard error of ±1 °C.

## Data Availability

The data presented in this study are available in this article.

## Supplementary Materials

The following supporting information can be downloaded at https://www.mdpi.com/article/10.3390/molecules29112420/s1: Figure S1: The catalytic reactions of 8-17 DNAzymes in the cell-mimicking buffer; Figure S2: DNAzymes with extra W^12.0^ evaluated under single-turnover conditions in the presence of 10 μM metal ions; Figure S3: The catalytic reactions of DNAzymes with extra W^12.0^ in the presence of higher concentrations of metal ions.
