# Supplementary material for: The Programmable Catalytic Core of 8-17 DNAzymes
Source: Molecules. 2024 May 21;29(11):2420. doi: 10.3390/molecules29112420 (PMC11173380; doi:10.3390/molecules29112420)
Supplement: Supplementary file 1 [file molecules-29-02420-s001.zip › molecules-3008876-supplementary.pdf]

Electronic supplementary materials

The Programmable Catalytic Core of 8-17 DNAzymes

Fumei Zhang, Weiguo Shi, Lei Guo, Shihui Liu, Junlin He

Content

Figure S1 The catalytic reactions of 8-17 DNAzymes in the cell-mimicking buffer S2
Figure S2 DNAzymes with extra W12.0 evaluated under single-turnvover conditions in the presence of 10 μM metal ions. S2
Figure S3 The catalytic reactions of DNAzymes with extra W12.0 in the presence of higher concentrations of metal ions. S3

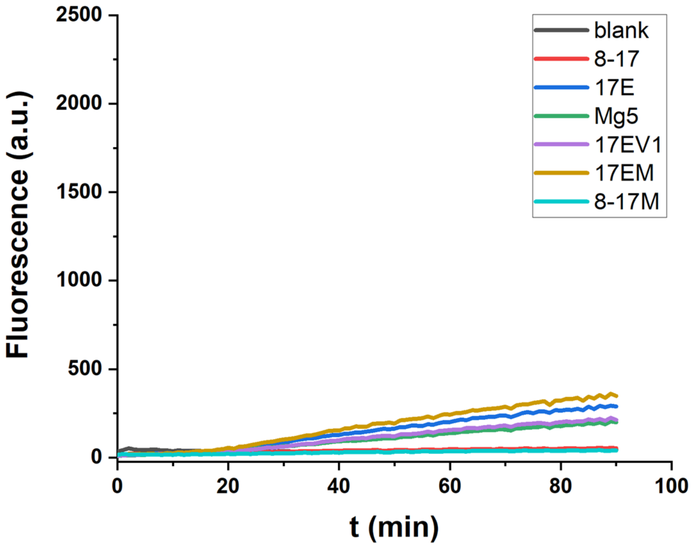

Figure S1 The catalytic reactions of 8-17 DNAzymes in the cell-mimicking buffer. The buffer containing 100 mM NaCl, 50 mM KCl, and 1 mM $MgCl_2$ in 50 mM Tris-HCl (pH 7.40) was used, under the single-turnover conditions (0.5 μM DNAzyme versus 0.05 μM substrate).

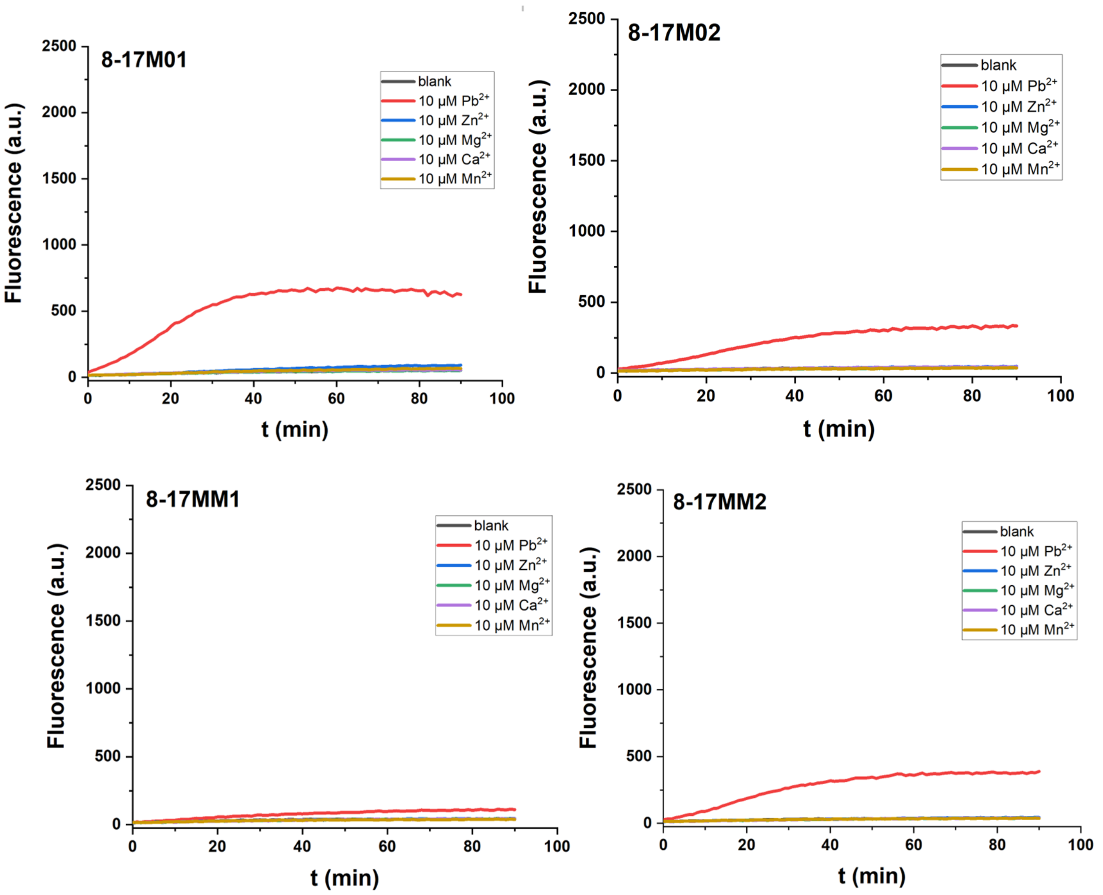

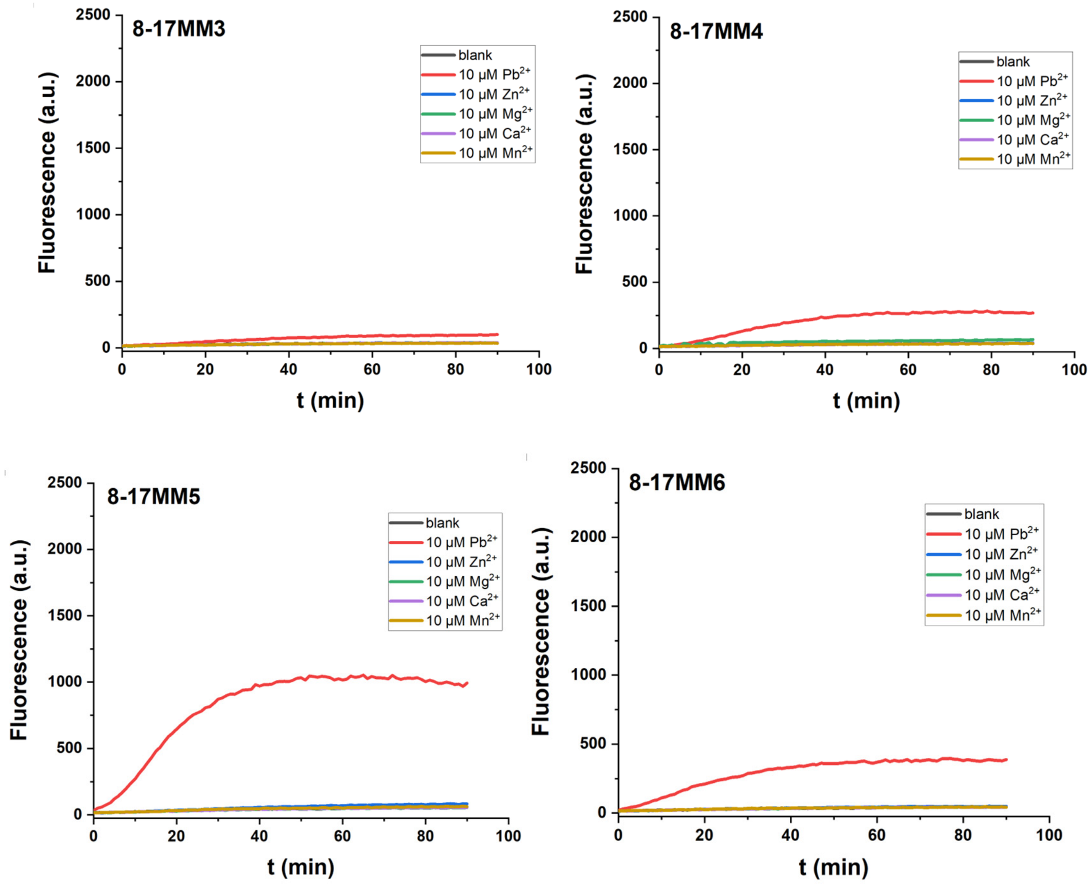

Figure S2 DNAzymes with extra $W^{12.0}$ evaluated under single-turnover conditions in the presence of 10 μM metal ions. The reaction between 0.5 μM DNAzyme and 0.05 μM substrate was conducted in the buffer (50 mM HEPES, pH 7.27), in the presence of 10 μM Ca^{2+}, Mg^{2+}, Zn^{2+}, Pb^{2+}, Mn^{2+}, respectively.

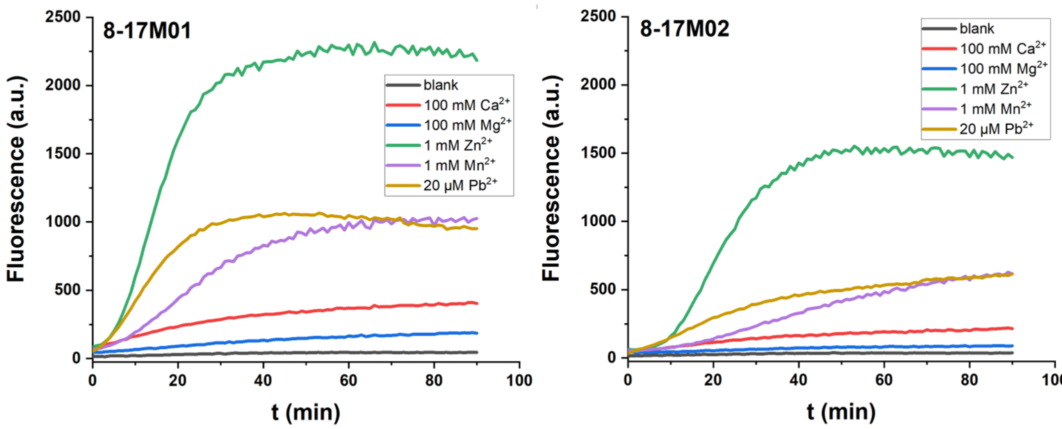

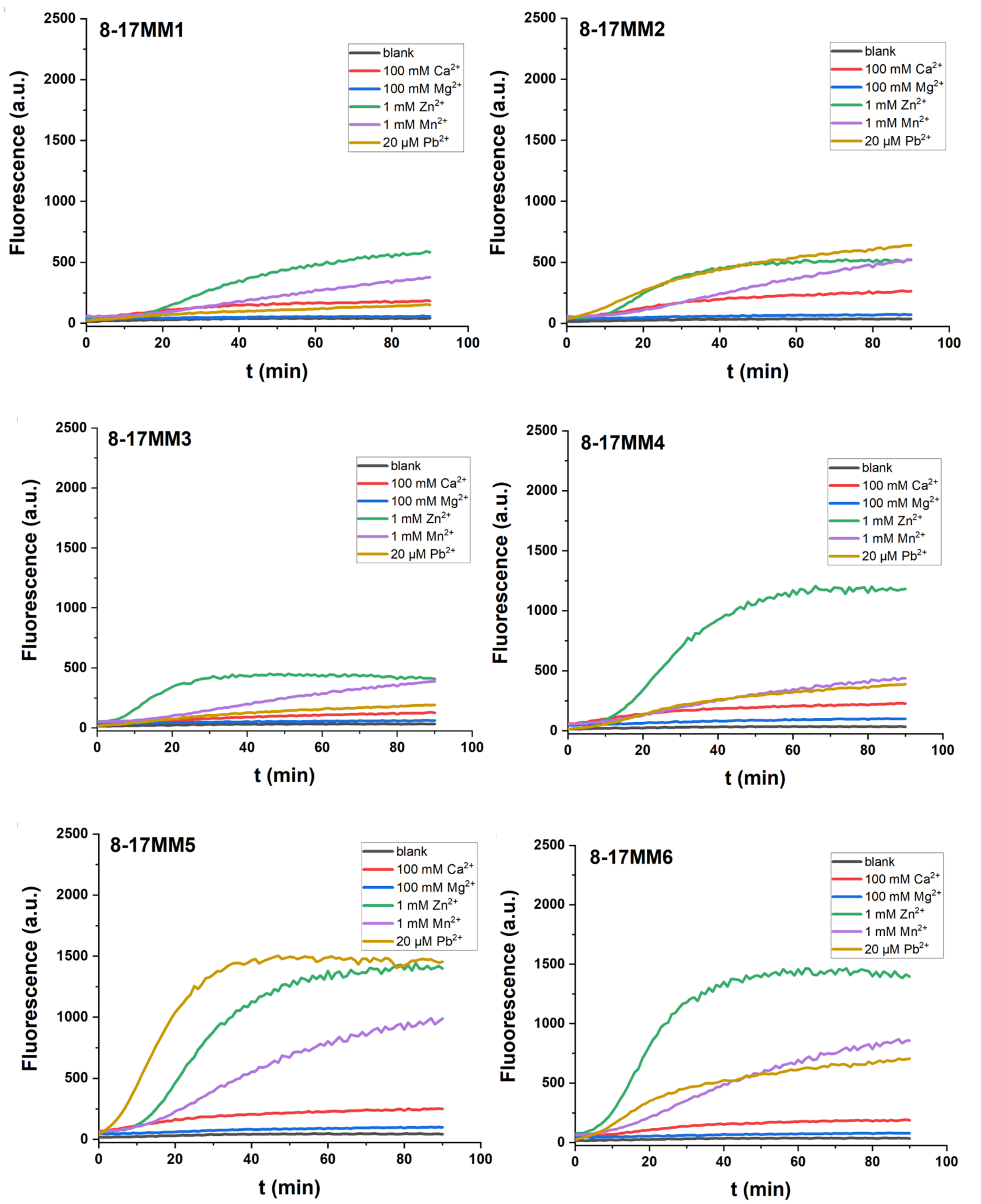

Figure S3 The catalytic reactions of DNAzymes with extra $W^{12.0}$ in the presence of higher concentrations of metal ions. Single-turnover conditions were used: 0.5 μM DNAzyme and 0.05 μM substrate in the buffer (50 mM HEPES, pH 7.27) containing 100 mM Na^{+}, in the presence of Ca^{2+} (1 mM), Mg^{2+} (1 mM), Mn^{2+} (1 mM), Zn^{2+} (250 μM), Pb^{2+} (20 μM), respectively.
